# Influence of Size and Shape of Silica Supports on the Sol–Gel Surface Molecularly Imprinted Polymers for Selective Adsorption of Gossypol

**DOI:** 10.3390/ma11050777

**Published:** 2018-05-11

**Authors:** Keke Zhi, Lulu Wang, Yagang Zhang, Yingfang Jiang, Letao Zhang, Akram Yasin

**Affiliations:** 1 Xinjiang Technical Institute of Physics and Chemistry, Chinese Academy of Sciences, Urumqi 830011, China; zhikeke@ms.xjb.ac.cn (K.Z.); wanglulu@ms.xjb.ac.cn (L.W.); jiangyf@ms.xjb.ac.cn (Y.J.); zhanglt@ms.xjb.ac.cn (L.Z.); akram@ms.xjb.ac.cn (A.Y.); 2 University of Chinese Academy of Sciences, Beijing 100049, China; 3 Department of Chemical and Environmental Engineering, Xinjiang Institute of Engineering, Urumqi 830026, China

**Keywords:** gossypol, surface molecular imprinting, size and shape effect, sol–gel process

## Abstract

The influence of various silica gel supports with different shapes and sizes on the recognition properties of surface molecular imprinted polymers (MIPs) was investigated. MIPs for selective recognition and adsorption of gossypol were synthesized via the sol–gel process with a surface imprinting technique on silica gel substrates. 3-aminopropyltriethoxysilane (APTES) and tetraethoxysilane (TEOS) were chosen as the functional monomer and the cross-linker. The morphology and structure of the gossypol-MIPs were characterized using Fourier transform infrared spectroscopy (FT-IR), scanning electron microscopy (SEM), and a standard Brunauer–Emett–Teller (BET) analysis. Results indicated that the surface imprinted polymer layer facilitated the removal and rebinding of the template, and thus, achieved fast binding kinetics. Compared with the MIPs prepared on irregularly shaped silica with a broad particle size distribution, the MIPs using regularly-shaped silica of uniform size showed higher imprinting factor (IF), and the MIP made with a relatively larger sized (60 μm) spherical silica, demonstrated higher adsorption capacity compared to the MIPs made with smaller sized, spherical silica. The MIP prepared with 60 μm spherically shaped silica, featured a fast adsorption kinetic of 10 min, and a saturated adsorption capacity of 204 mg·g^−1^. The gossypol-MIP had higher selectivity (IF = 2.20) for gossypol over its structurally-similar analogs ellagic acid (IF = 1.13) and quercetin (IF = 1.20). The adsorption data of the MIP correlated well with the pseudo-second-order kinetic model and the Freundlich isotherm model, which implied that chemical adsorption dominated, and that multilayer adsorption occurred. Furthermore, the MIP exhibited an excellent regeneration performance, and the adsorption capacity of the MIP for gossypol only decreased by 6% after six reused cycles, indicating good application potential for selective adsorption of gossypol.

## 1. Introduction

The molecular imprinting technique is an attractive approach for molecular recognition with high affinity and selectivity [1,2,3,4,5]. This technology has been extensively investigated in various areas, such as stationary phase extractions, sensors, chromatographic separation, and as a mimic for enzyme catalysis [6,7,8,9,10,11]. Three-dimensional binding cavities with shapes, sizes, and functional groups complementary to the template molecule, were produced during the synthesis process of MIPs, thereby enabling the re-identification of the target molecules with preference over other structurally-similar analogs. MIPs are commonly prepared using bulk polymerization [12]. The main disadvantages of this approach are poor site accessibility for target molecules, and the potential for trapped templates to generate “dead” sites, which result in incomplete template removal, and slow binding kinetics, as well as bleeding of the template. In order to address this issue, a surface molecular imprinting technique was developed [13], which allows the imprinted cavities on the surface of supports, and, thus, can greatly improve the accessibility of the imprinting sites and enhance binding kinetics [14,15]. Silica gel particles, because of their high stability, good biocompatibility, non-swelling property and low-cost, have been considered to be ideal support material for surface molecular imprinting [16,17,18,19]. However, to the best of our knowledge, the influence of shape and size of silica support on the recognition properties of the resulting MIPs, have rarely been reported. This study will lead to an in-depth understanding of the structure-property relationship in the recognition process of surface molecular imprinting.

Recently, molecularly imprinted sol–gel materials have also been reported [20,21,22,23,24,25]. These materials were synthesized via a convenient process, to incorporate the template molecules into rigid inorganic–organic matrices. Compared with acrylic-based MIPs, the sol–gel MIPs have a limited number of functional monomers. For example, there are no commercially available silane agents with carboxylic acid functional groups, and the recipes of TEOS and APTES are mostly used. The advantages of sol–gel MIPs are mild reaction conditions and simple operation [26]. This work aims to develop a kind of MIP combining the advantages of surface imprinting and sol−gel technology.

Cotton is one of the most important agricultural crops. The United States and China are two of the largest cotton producers worldwide. Gossypol is a toxic polyphenolic compound extracted from cotton plants [27,28]. Its main toxicities include growth depression, reproductive diseases, and intestinal and internal organ abnormalities in mammals [29,30,31]. Cottonseed meal, a by-product of cottonseed oil, is becoming an indispensable part of protein feed resources for animal husbandry and aquaculture, because of its high nutritional quality and low cost. However, the presence of poisonous gossypol severely restricts its application. Thus, removal of gossypol in cottonseed products has become significantly important.

For this purpose, various approaches have been developed, including ferrous sulfate treatment [32], solvent extraction [33], microbial fermentation [34], and adsorption treatment [35,36,37]. Among those, adsorption is a promising approach owing to its mild operation conditions and low-cost. Alumina, silica, synthetic magnesium silicates, and MIPs [35,36,37], have been reported as adsorbents for the removal of gossypol. In comparison to other adsorbents, MIPs possess unique advantages of high selectivity and efficiency. Our research group has reported the MIP for adsorption of gossypol, by bulk free radical polymerization [37]. To the best of our knowledge, silica-supported sol–gel surface imprinted polymers for gossypol have rarely been reported.

Along these lines, in the work reported here, MIPs for the selective recognition and adsorption of gossypol were designed by combining a sol–gel process with a surface imprinting technique on silica gel supports. Importantly, the influence of the shapes and sizes of silica gel supporting materials on the recognition properties of the surface gossypol imprinted polymers, were systematically investigated for the first time. The superiority of silica gel with a larger particle size and regular spherical shape, over other kinds of silica gel in preparing MIP, were then illustrated. The performance of the MIPs in the selective recognition and adsorption of gossypol, was evaluated using a solution adsorption experiment. The adsorption kinetics, adsorption capacity, adsorption isotherms, adsorption selectivity, as well as the reusability, were discussed in detail as well.

## 2. Materials and Methods

### 2.1. Materials and Reagents

Six different kinds of silica gel were chosen as supports to prepare the gossypol surface imprinted polymers. Silica spheres (0.5 μm average particle size) were obtained from Alfa Aesar (Shanghai, China); silica spheres (5 μm average particle size) were obtained from Fuji Silysia Chemical Ltd. (Aichi, Japan); silica spheres (60 μm average particle size) were obtained from Acchrom Technologies Co., Ltd. (Beijing, China); irregularly shaped silica gel (Thin layer chromatography, 10–40 μm), (200–300 meshes, 50–75 μm particle size) and (100–200 meshes, 75–150 μm particle size) were all purchased from Qingdao Haiyang Chemical Co., Ltd. (Qingdao, China). Gossypol was purchased from Sigma-Aldrich (Shanghai, China); (3-aminopropyl)triethoxysilane (APTES) (98%) was purchased from Alfa Aesar (Shanghai, China); tetraethoxysilane (TEOS) (98%), Quercetin (99%) and ellagic acid (98%) were purchased from Adamas (Shanghai, China); methanol, acetone, acetic acid, hydrochloric acid, and sodium hydroxide were all analytical grade, and were purchased from Tianjin Zhiyuan Chemical Co., Ltd. (Tianjin, China). Water was purified using a Millipore Milli-Q gradient system (Billeria, MA, USA) to high-performance liquid chromatography (HPLC) grade.

### 2.2. Preparation of Surface MIPs

A typical procedure for the synthesis of MIPs was as follows. Silica (8.0 g) and 60 mL of 33% (w/w) methanesulfonic acid aqueous solution were mixed in a 250 mL flask and refluxed for 8 h at 110 °C under stirring. After that, the resultant silica was washed with pure water to a neutral pH, and the silica particles were separated from solution by centrifugation. Then, they were dried under a vacuum at 70 °C for 10 h, and the activated silica particles were obtained [38].

Gossypol (300 mg, 0.57 mmol) was first dissolved in 20 mL acetone. Then, APTES (2.0 mL, 8.5 mmol) was added, followed by stirring for 30 min to form monomer-template complex. After that, activated silica gel (400 mg) was added while stirring for 30 min to ensure silica particles were fully dispersed. Finally, TEOS (4.0 mL, 18 mmol) and acetic acid (1.0 mL of 1.0 mol·L^−1^) were added to the mixture while stirring at room temperature for 24 h to obtain highly cross-linking polymer particles. The non-imprinted polymers (NIPs) were also prepared through the same procedure, without adding template gossypol [39].

To remove gossypol, the obtained polymer was repeatedly washed with 50 mL mixture of methanol and 6 mol·L^−1^ HCl (1:1, v/v) until no template molecules were detected by a UV-Vis spectroscopy. Then, they were neutralized with 0.1 mol·L^−1^ NaOH solution and washed with ultra pure water and methanol. For NIPs, the particles were washed to a neutral pH with ultra pure water and methanol to remove the un-reacted substances. Finally, both MIPs and NIPs were dried at 80 °C for 12 h in a vacuum drying oven.

### 2.3. Characterization

The binding capacity of the MIPs and NIPs were measured by UV-Vis spectroscopy (UV-2600, Shimadzu, Kyoto, Japan). The morphologies of the samples were measured using field emission scanning electron microscopy (FESEM) (SUPRA 55VP, Zeiss, Oberkochen, Germany). Fourier transform infrared (FT-IR) spectra were collected with a Bruker VERTEX-70 spectrometer (Bruker, Karlsruhe, Germany) in the range of 4000–500 cm^−1^ using KBr pellets. Nitrogen adsorption–desorption measurements were performed on an automatic adsorption instrument (Autosorb-IQ, Quantachrome Instrument Corp., Boynton Beach, FL, USA) and the specific surface areas (S) were calculated using the BET method.

### 2.4. Binding Experiments

Solution binding studies were carried out to investigate adsorption performance of MIPs and NIPs for gossypol. In this study, the adsorption of gossypol was measured by UV-Vis spectrometry, and the typical UV absorption peak of gossypol at 373 nm was used for the quantitative analysis in the experiments.

In the kinetic adsorption experiments, 10 mg of MIPs or NIPs were added to 5.0 mL of gossypol solution in methanol at an initial concentration (C_0_, mg·L^−1^) of 110 mg·L^−1^, and were shaken at time intervals from 0 min to 60 min. Then, the supernatants and polymers were separated using syringe filters (0.2 μm, PTFE) and the concentrations of gossypol in the supernatant (C_t_, mg·L^−1^) were measured by UV-Vis spectrometry following a reported procedure [37]. The adsorption capacity (Q_t_, mg·g^−1^) at given times can be calculated according to Equation (1)

(1) $$Q_{t}=\frac{\left(C_{0}-C_{t}\right)V}{m}$$

where V (L) is the volume of gossypol solution, and m (g) is the weight of the absorbents.

In the adsorption isothermal experiments, 10 mg of MIPs or NIPs were added to 5 mL of gossypol solutions in methanol at various concentrations from 110 mg·L^−1^ to 1600 mg·L^−1^ and were shaken for 6 h to ensure the adsorption equilibrium could be reached. The supernatants and polymers were separated by a syringe filter, and the supernatants were analyzed to determine the remaining concentration (C_e_, mg·L^−1^) using UV-Vis spectrometry [37]. The equilibrium adsorption capacity (Q_e_, mg·g^−1^) was calculated according to Equation (2).

(2) $$Q_{e}=\frac{\left(C_{0}-C_{e}\right)V}{m}$$

In the adsorption selectivity experiments, two structurally-similar analogs of ellagic acid and quercetin were selected. 10 mg of MIPs or NIPs were added to 5 mL of each methanol solution of 200 mg·L^−1^ of gossypol, ellagic acid and quercetin solution in methanol. After being shaken for 1 h, the supernatants and polymers were separated, and the residual concentrations of gossypol, ellagic acid and quercetin were measured by UV-Vis spectrometry at 374, 366, and 371 nm, respectively. Additionally, the imprinting factor (IF) and selectivity coefficients (α) were used to evaluate the recognition and selectivity of MIPs and NIPs towards gossypol and competitive compounds. IF and α were calculated from Equations (3) and (4)

(3) $$\mathrm{IF}=\frac{Q_{\mathrm{MIP}}}{Q_{\mathrm{NIP}}}$$

(4) $$\alpha =\frac{{\mathrm{IF}}_{T}}{{\mathrm{IF}}_{C}}$$

where Q_MIP_ (mg·g^−1^) and Q_NIP_ (mg·g^−1^) represent the adsorption capacity of the templates, or analogs on MIPs and NIPs at the same conditions, respectively. IF_T_ and IF_C_ were the imprinting factors for gossypol and contrastive compounds, including ellagic acid and quercetin, respectively.

### 2.5. Reusability of MIPs

The reusability of MIPs was investigated through six adsorption−desorption cycles. 10 mg of MIPs were added to 5.0 mL of gossypol solution in methanol at a concentration of 200 mg·L^−1^ and was incubated for 1 h. After adsorption, the MIPs saturated with gossypol were collected by centrifugation, and were washed with a mixture of methanol and 6 mol·L^−1^ HCl (1:1, v/v) to remove gossypol completely, washed with ultra pure water to a neutral pH, and dried under vacuum. Then, the recovered MIPs were reused for rebinding of gossypol.

## 3. Results and Discussion

### 3.1. Preparation of the Gossypol-MIPs 

**Figure 1:**
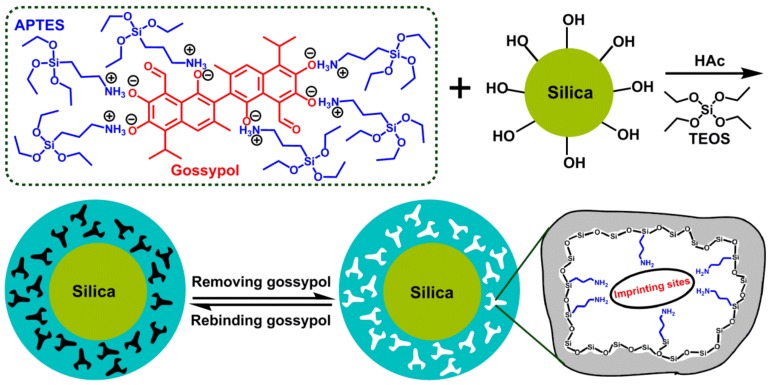
Synthetic route of gossypol-MIPs by combining sol–gel strategy and surface imprinting technique.

The schematic procedure for the synthesis of gossypol-MIPs was illustrated in Figure 1. Our approach combined the surface imprinting and sol–gel process. Because commercial silica gel had a low concentration of surface silanol groups suitable for modification, the activation of silica gel surface is necessary. The silica was first activated with methanesulfonic acid aqueous solution by the rationale of rehydroxylation and in favor of the formation of the imprinted silica coating. The template gossypol (value of pKa about 6.5 [40]) which has six acidic hydroxyl –OH groups, and the functional monomer APTES which has basic amino –NH_2_ group, formed a complex through acid−base ionic pair interaction. APTES was not only the functional monomer, but also the intermediate to link silica particles [39]. Then, the complex was anchored onto the silica surface of silica by hydrolysis, and condensation of TEOS and APTES in the presence of HAc catalyst. Thus, the rigid polymeric network was formed. Finally, after removal of the template, an imprinted polymer layer with a large number of tailor-made imprinted cavities complementary to gossypol in shape, size, and functional group, was obtained on the surface of silica. There were acid-catalyzed hydrolysis and base-catalyzed hydrolysis for the sol–gel process. Under low pH conditions, condensation occurred preferentially between silanol groups located on monomers or at the ends of polymers, and when the gels were composed of predominantly entangled linear chains. Under high pH conditions, condensation preferentially occurred between the more highly branched oligomers, to form more particulate gels. Gossypol as the template molecule was very sensitive to alkaline substance, including ammonia hydroxide, so acetic acid was chosen as the catalyst. In the preparation process, the amount of template molecule gossypol was 0.57 mmol, and the amount of functional monomer, APTES, used was 8.5 mmol. The gossypol to APTES ratio was 1:15. In molecular imprinting, the template to monomer ratio is often 1:8 to 1:12. An excess of functional monomer is always used in order to push the equilibrium to the right, promoting the formation of high affinity imprinting sites. For the gossypol molecule, it contains six phenolic hydroxyl functional groups in its structure, which can interact with six amino groups of the monomer. Thus, the ratio of gossypol to APTES is favorable. The TEOS amount is 14 mmol, and the TEOS to APTES ratio is 1.6:1, which is also reasonable in order to form a crosslinked network. The amounts of APTES and TEOS determine the thickness of the imprinted layer, and the thickness of the sol−gel imprinted layer on the surface of silica increases with increasing amounts of APTES and TEOS, making a larger sized MIP layer.

### 3.2. Characterization of MIPs 

#### 3.2.1. FTIR Analysis

**Figure 2:**
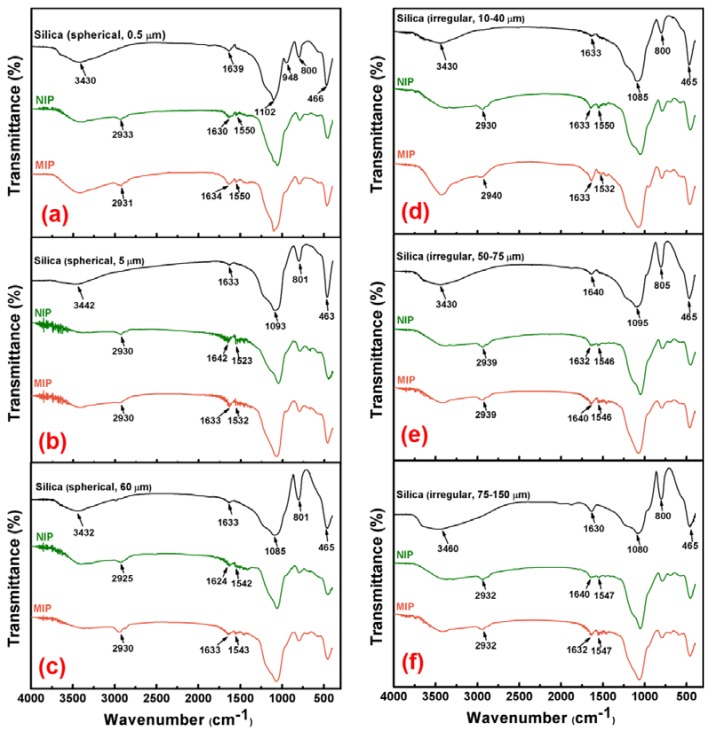
FT-IR spectra of silica support and its corresponding NIP and MIP. (**a**) spherical 0.5 μm, (**b**) spherical 5 μm, (**c**) spherical 60 μm, (**d**) irregular 10–40 μm, (**e**) irregular 50–75 μm, and (**f**) irregular 75–150 μm.

The FTIR spectra of the silica supports, and the corresponding NIPs and MIPs, were illustrated in Figure 2, which provided direct evidence for the successful preparation of MIPs. As shown in Figure 2a, silica spheres of 0.5 μm size, showed the bands around 3430 cm^−1^ and 1102 cm^−1^, indicating –OH and Si–O–Si vibration. The peaks around 800 cm^−1^ and 466 cm^−1^ were attributed to Si–OH and Si–O stretching vibrations [41], respectively. For IR of the corresponding MIP, the bands at 1550 cm^−1^ and 1634 cm^−1^ represented N–H bond, and the 2933 cm^−1^ signal was associated to C–H bond. Moreover, it was found that there were no obvious differences between the spectra of the MIP and NIP. For the spectra of other samples in Figure 2b–f, the characteristic signals corresponding to different functional groups, were all marked. They exhibited similar locations, appearances and intensities of the major bands compared with the bands in Figure 2a, which confirmed the fact that they had similar backbones. These results suggest that APTES had been successfully grafted on the surface of silica support after imprinting.

#### 3.2.2. SEM Analysis

**Figure 3:**
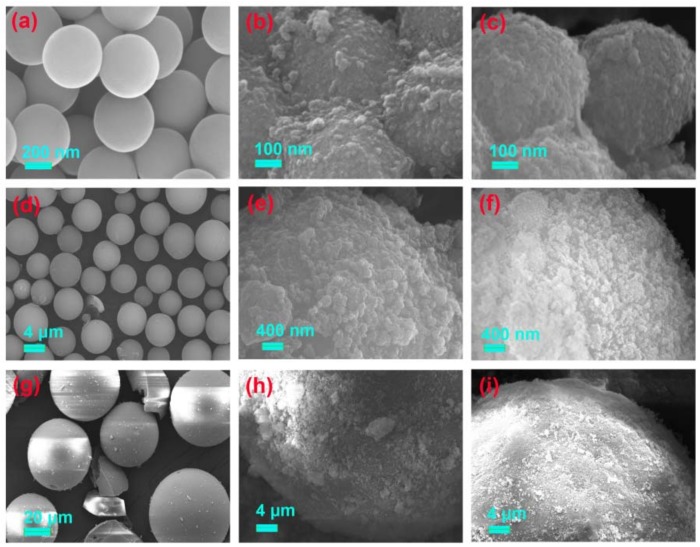
SEM images of silica support and its corresponding NIP and MIP (**a**) spherical 0.5 μm silica, (**b**) spherical 0.5 μm silica-NIP and (**c**) spherical 0.5 μm silica-MIP; (**d**) spherical 5 μm silica, (**e**) spherical 5 μm silica-NIP and (**f**) spherical 5 μm silica-MIP; (**g**) spherical 60 μm silica, (**h**) spherical 60 μm silica-NIP and (**i**) spherical 60 μm silica-MIP.

**Figure 4:**
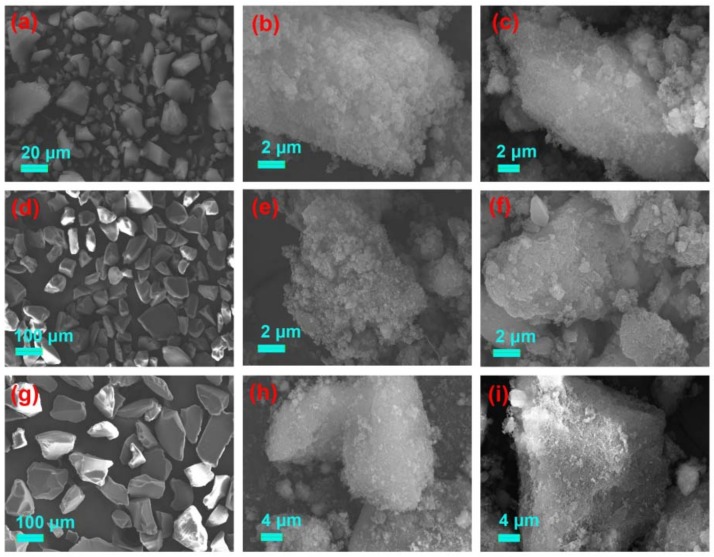
SEM images of silica support and its corresponding NIP and MIP (**a**) irregular 10–40 μm silica, (**b**) irregular 10–40 μm silica-NIP and (**c**) irregular 10–40 μm silica-MIP; (**d**) irregular 50–75 μm silica, (**e**) irregular 50–75 μm silica-NIP and (**f**) irregular 50–75 μm silica-MIP; (**g**) irregular 75–150 μm silica, (**h**) irregular 75–150 μm silica-NIP and (**i**) irregular 75–150 μm silica-MIP.

The surface morphologies of the six activated silica gel particles and their MIPs and NIPs, are shown in Figure 3 and Figure 4. The difference between the silica supports and corresponding NIP and MIP, was obvious. All silica supports displayed a smooth surface. However, the gossypol-MIPs all had highly rough surfaces and larger particle sizes, indicating the formation of imprinted coating layers and the successful preparation of gossypol-MIPs. Additionally, the size of the resultant MIP particles changed considerably following the imprinting process. From the SEM images in Figure 3c, the size of MIP particles increased to 900 nm, compared to its corresponding 0.5 μm bare silica particles in Figure 3a, which indicated a thickness of 200 nm for the imprinted layer coated on the surface of the silica. It could also be seen that the size of MIP in Figure 3f,i prepared on the 5 μm in Figure 3d, and 60 μm in Figure 3g, bare silica particles also increased. Additionally, the size of MIP in Figure 4c,f,i on the surface of irregular silica, all increased compared to its corresponding 10–40 μm, 50–75 μm, and 75–150 μm bare silica particles in Figure 4a,d,g. Furthermore, the surface morphology of MIPs was not distinctively different from their NIPs. This observation provided good evidence that the distinct binding property between MIPs and NIPs was not due to the surface morphology change, but indeed the molecular imprinting process.

#### 3.2.3. Standard BET Analysis

MIPs were characterized with the nitrogen adsorption-desorption analysis. The specific surface area (S) was calculated using Multi-Point Brunauer–Emett–Teller (BET) methods, the total pore volume was estimated using a single point method at P/P_0_ = 0.993, and the average pore diameter was obtained by Barret–Joyner–Halenda (BJH) method. Table 1 lists the structure parameters of all MIPs, NIPs and their silica supports, including BET surface area, total pore volume and average pore diameter. It was found that the specific surface area for 0.5 μm spherical silica was 14.29 m^2^·g^−1^, which was much smaller than all other five silica gels, including 5 μm spherical silica (280.9 m^2^·g^−1^), 60 μm spherical silica (431.4 m^2^·g^−1^), 10–40 μm irregular silica (299.9 m^2^·g^−1^), 50–75 μm irregular silica (275.9 m^2^·g^−1^) and 75–150 μm irregular silica (252.2 m^2^·g^−1^). The number of pores on the particle also affected the specific surface area. The more pores, the larger the specific surface area. The pore volume of 0.5 μm silica was 0.08242 cm^3^·g^−1^, and the pore volume of 5 μm silica was 0.8205 cm^3^·g^−1^, which was 10 times larger than 0.5 μm silica. The larger pore volume resulted from the particle having many more pores. Therefore, it was not difficult to understand that the specific surface area for the 0.5 μm silica was smaller than the other silica particles.

In comparison to silica support, for example, 0.5 μm spherical silica (14.3 m^2^·g^−1^), there was a significant increase of surface area and pore volume for its corresponding MIP (268.2 m^2^·g^−1^, 0.3716 cm^3^·g^−1^) and NIP (31.53 m^2^·g^−1^, 0.1757 cm^3^·g^−1^). This could be due to the formation of the porous surface imprinting layer on silica support. Noticeably, for other larger silica support, there was a decrease in surface area and pore volume for the corresponding MIPs and NIPs. For example, the specific surface area and pore volume value of the 5 μm spherical silica (280.9 m^2^·g^−1^, 0.8205 cm^3^·g^−1^) were higher than its MIP (124.7 m^2^·g^−1^, 0.2206 cm^3^·g^−1^) and NIP (52.75 m^2^·g^−1^, 0.1380 cm^3^·g^−1^). Furthermore, all the MIPs showed higher specific surface areas and pore volume than the corresponding NIPs. This implied that the imprinting process helped form more cavities and pores [42]. For example, the specific surface area and pore volume of MIP prepared on the 60 μm spherical silica, were 112.2 m^2^·g^−1^ and 0.3158 cm^3^·g^−1^, which were 1.5 times and 1.8 times of its corresponding NIP (73.14 m^2^·g^−1^, 0.1755 cm^3^·g^−1^), respectively. The results were consistent with the fact that MIP normally showed larger surface area and pore volume than the non-imprinted material [22,43]. In addition, the data revealed that all MIPs showed mesoporous (2–50 nm) type characteristics, which were more desirable sorbents for application of solid phase extraction than micropores or macropores, due to good solvent permeability and easy diffusion of target analytes [44].

### 3.3. Adsorption Kinetics of MIPs 

The adsorption rate is an important parameter for the adsorption process. As shown in Table 2, the MIPs prepared by a surface imprinting technique all demonstrated rapid adsorption kinetics, with the adsorption equilibrium time less than 50 min. The imprinting factor (IF) was used to evaluate the imprinting effect of MIPs towards the template. Results showed that the IF values of the polymers prepared on regular spherical silica with uniform size, were all larger than those prepared on the silica with an irregular shape and broad particle size distribution [15]. This was probably due to the fact that the curved surface and regular shape helped form uniform coatings. The irregular shape and broad particle size distribution was not ideal for the imprinting. The equilibrium adsorption capacities of all samples are listed in Table 2. The MIP prepared on 60 μm spherical silica, had much higher binding capacity (92.9 mg·g^−1^) than the MIP (65.5 mg·g^−1^) made with spherical 0.5 μm silica, and MIP (51.4 mg·g^−1^) made with spherical 5 μm silica. Based on the imprinting factor and binding capacity data, 60 μm spherical silica was found to be superior support for preparing gossypol MIP and was selected for the subsequent experiments.

**Figure 5:**
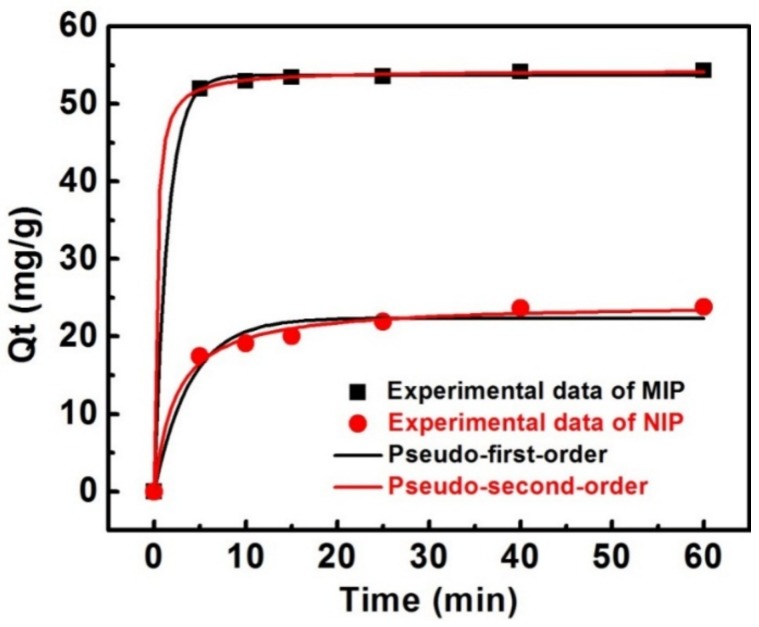
Adsorption kinetics of gossypol binding onto MIPs and NIPs prepared using spherical silica 60 μm as the support (10 mg of absorbents in 5.0 mL of 110 mg·L^−1^ gossypol solution).

The adsorption performance of MIP prepared using 60 μm spherical silica was further studied in consideration of its outstanding adsorption capacity and excellent imprinting effect. Figure 5 illustrates the adsorption profiles of the MIP and NIP for gossypol over time. The adsorption capacity increased rapidly during the first 5 min and then the curve leveled off as equilibrium was reached within 10 min. Conversely, gossypol adsorption for NIP reached equilibrium at 20 min. The MIP displayed faster adsorption kinetics than the NIP. This could be attributed to more desirable imprinting cavities generated in the surface imprinting polymer layer.

In order to probe the rate-determining step, pseudo-first-order (Equation (5)) and pseudo-second-order kinetic model [45,46] (Equation (6)), were employed to simulate the binding procedure,

(5) $$\ln\left(Q_{e}-Q_{t}\right)=\ln Q_{e}-k_{1}t$$

(6) $$\frac{t}{Q_{t}}=\frac{1}{k_{2}Q_{e}^{2}}+\frac{t}{Q_{e}}$$

where Q_e_ (mg·g^−1^) and Q_t_ (mg·g^−1^) are the binding capacity of gossypol adsorbed at equilibrium and at time t (min), k_1_ (min^−1^) and k_2_ (g·mg^−1^·min^−1^) are the rate constant of pseudo-first-order and pseudo-second-order adsorption model, respectively.

The parameters of adsorption kinetics were shown in Table 3 and the nonlinear regression plots of the two models were shown in Figure 5. The applicability of the kinetic models to the adsorption behaviors was evaluated by the correlation coefficients (R^2^). It was found that the R^2^ values of the MIP and NIP by pseudo-second-order kinetic model, both exceeded 0.99, and the R^2^ value for MIP was as high as 0.9999. Moreover, the theoretical adsorption capacity (Q_e,cal_, 54.30 mg·g^−1^) estimated from the pseudo-second-order kinetic model, perfectly agreed with the experimental adsorption data (Q_e,exp_, 54.3 mg·g^−1^). The adsorption kinetic process perfectly fits the pseudo-second-order model, indicating that the chemisorption could be the rate-limiting step controlling the gossypol–binding process, and that the adsorption capacity was proportional to the number of active binding sites on the surface of the MIP [47].

### 3.4. Adsorption Isotherms of MIPs

**Figure 6:**
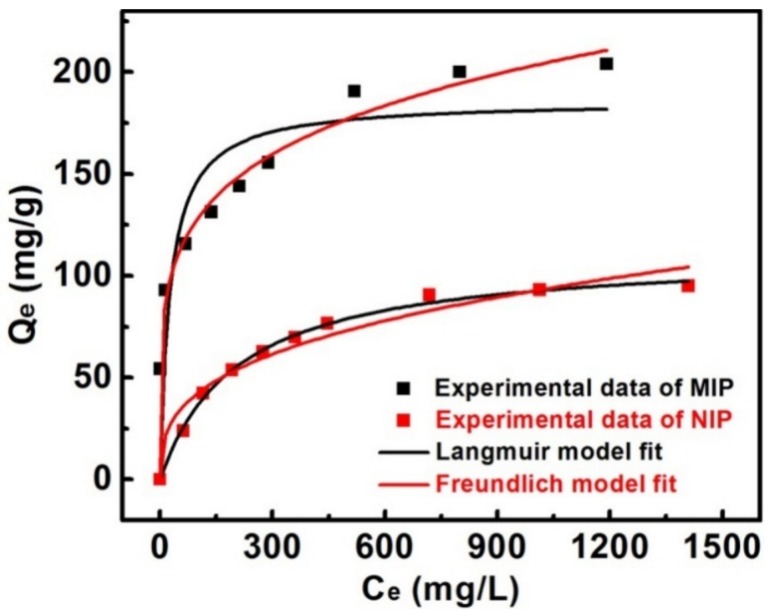
Adsorption isotherms of gossypol binding onto MIP and NIP prepared on the spherical silica 60 μm (10 mg of absorbents in 5.0 mL gossypol solution for 6 h).

To estimate the adsorption capability of the MIPs and NIPs, adsorption isotherm experiments were conducted with different initial concentrations of gossypol solution. Figure 6 shows that the adsorption capacity of the MIP or NIP prepared on the 60 μm spherical silica, increased with the gossypol concentration. This might be ascribed to the increasing driving force of the concentration gradient which could accelerate the diffusion of gossypol into MIP matrix. Moreover, the amount of gossypol bound to the MIP was much higher than that of NIP, and the saturation adsorption capacity of MIP was as high as 204 mg·g^−1^. This indicated that the formation of the surface imprinted layer had high recognition ability and excellent imprinting effect for gossypol. The results also implied that the imprinted process formed the specific recognition sites for gossypol in the MIP matrix.

Langmuir (Equation (7)) and Freundlich (Equation (8)) isotherm models [48,49] are widely used for describing the experimental data of adsorption isotherms. The Langmuir model assumes that the adsorption took place on a homogeneous surface with monolayer coverage. The Freundlich isotherm model was an exponential equation that described reversible adsorption. It was suitable for multilayer adsorption of a heterogeneous system and was not restricted to the formation of the monolayer. They can be expressed respectively as follows:

(7) $$Q_{e}=\frac{Q_{m}K_{L}C_{e}}{1+K_{L}C_{e}}$$

(8) $$Q_{e}=K_{F}C_{e}^{1/n}$$

where Q_e_ (mg·g^−1^) and C_e_ (mg·L^−1^) are adsorption capacity and free concentration of gossypol at adsorption equilibrium, respectively; Q_m_ (mg·g^−1^) represents the maximum adsorption capacity of the adsorbent. K_L_ (L·mg^−1^) is Langmuir constant, which is related to affinity of the binding sites. K_F_ (mg·g^−1^) and n are both Freundlich constants that represent the adsorption capacity and adsorption favorability of the system, respectively. If n > 1, this suggests favorable adsorption.

The nonlinear regression plots and the parameters fitted by two isotherm models are presented in Figure 6 and Table 4. It was found that the Freundlich model of gossypol binding onto MIP, and the Langmuir model of gossypol binding onto NIP, were well correlated with the experimental data (R^2^ > 0.99). This implied that the binding sites in the MIP were heterogeneous, and the adsorption of gossypol onto the NIP could be partially monolayer adsorption.

### 3.5. Adsorption Selectivity of the MIPs

**Figure 7:**
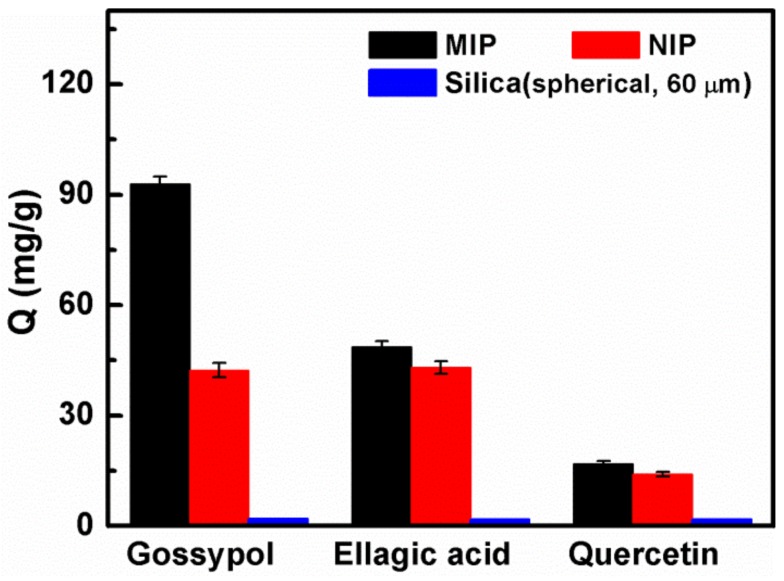
The selective adsorption capacity of the MIP, NIP and its silica support spherical (60 μm) toward gossypol, ellagic acid and quercetin solution, respectively (10 mg of absorbents in 5.0 mL 200 mg·L^−1^ gossypol solution for 1 h).

In order to evaluate the selectivity of the MIP for gossypol, two structurally similar compounds, ellagic acid and quercetin, were chosen as control templates. As shown in Figure 7, the adsorption capacity of the MIP prepared on 60 μm spherical silica for gossypol, was much higher than that of two analogues, meaning that the template gossypol had higher affinity than its competitive analogs for the imprinted materials. Indeed, the adsorption capacity of its NIP had no selectivity. The adsorption capacity of the bare silica support toward these three different compounds was all very low. Accordingly, the IF value listed in Table 5 of the polymers for gossypol was 2.2, which was higher than that of ellagic acid (IF = 1.13) and quercetin (IF = 1.20). The results indicated that the MIP exhibited high specificity for gossypol recognition. Furthermore, the selectivity coefficient values for gossypol, relative to ellagic acid and quercetin, were 1.94 and 1.83, respectively, indicating that the MIP had higher adsorption selectivity than that of the NIP. This was because selective recognition sites were absent in the NIP, and the adsorption is nonspecific. Therefore, it can be concluded that the MIP had outstanding specificity and high selectivity towards target gossypol.

### 3.6. Regeneration and Stability of the MIPs 

**Figure 8:**
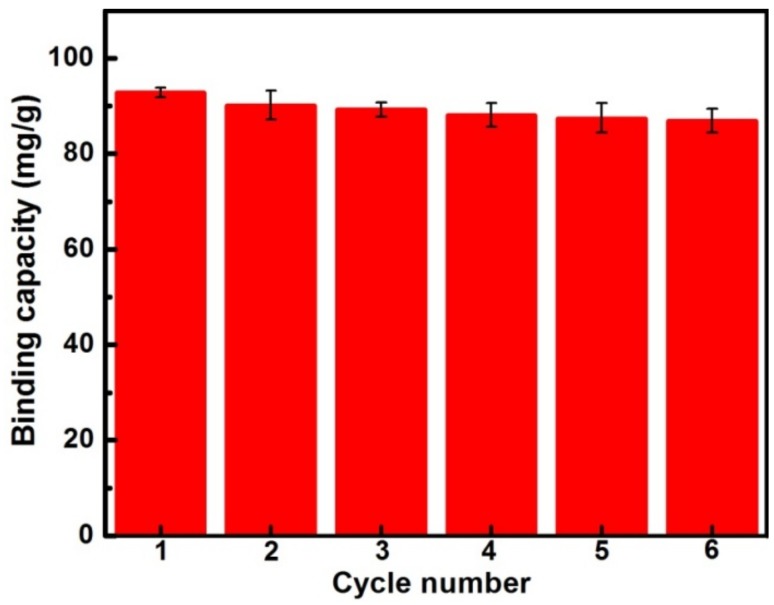
Reusability of MIP prepared with spherical silica (60 μm) for gossypol adsorption (10 mg of absorbents in 5.0 mL 200 mg·L^−1^ gossypol solution for 1 h).

As a promising gossypol adsorbent for future practical applications, regeneration potential and stability are vitally important. The used MIPs were recycled and reused for gossypol binding. As shown in Figure 8, MIP can be effectively regenerated. There was less than 6% loss of initial binding capacity after six cycles, indicating that MIP could be considered as an effective adsorbent of removing gossypol for recycled use without much of a decrease in adsorption capacity.

## 4. Conclusions

In this study, a series of gossypol-MIPs were prepared by combining sol–gel strategy and surface molecular imprinting technique, using silica gel with different shapes and sizes as the supports. All the MIPs exhibited fast binding kinetics. Results indicated that the surface imprinted polymer layer facilitated the removal and rebinding of the template, and thus, achieved fast binding kinetics. Compared to the MIPs prepared on the silica of irregular shape and a broad particle size distribution, the MIPs using silica of regular spherical shape and uniform size, showed higher imprint factors. MIPs made with relatively larger sized (60 μm) spherical silica, demonstrated higher adsorption capacity than the MIPs made with smaller sized spherical silica. The MIP prepared on the 60-μm spherical silica reached equilibrium at 10 min, and its adsorption kinetics closely followed the pseudo-second-order kinetic model. *Freundlich* isotherm model was well fitted for the adsorption isotherm data of gossypol. The maximum binding capacity of the MIP was 204 mg·g^−1^, which was two times higher than that of NIP. The gossypol-MIP had higher selectivity for gossypol over its structurally similar analogues, ellagic acid and quercetin. Furthermore, the MIP demonstrated good stability and regeneration performance.

## Figures and Tables

**Table 1:** BET surface area, pore volume and average pore diameter of six silica gel supports and their corresponding MIPs and NIPs.

Samples	BET Surface Area (m^2^·g^−1^)	Pore Volume (cm^3^·g^−1^)	Average Pore Diameter (nm)
Silica (spherical 0.5 μm)	14.29	0.08242	23.07
Spherical 0.5 μm NIP	31.53	0.1757	22.29
Spherical 0.5 μm MIP	268.2	0.3716	5.542
Silica (spherical 5 μm)	280.9	0.8205	11.68
spherical 5 μm NIP	52.75	0.138	10.47
spherical 5 μm MIP	124.7	0.2206	7.077
Silica (spherical 60 μm)	431.4	0.8606	7.981
spherical 60 μm NIP	73.14	0.1755	9.598
spherical 60 μm MIP	112.2	0.3158	11.26
Silica (irregular 10–40 μm)	299.9	0.9128	12.18
irregular 10–40 μm NIP	85.36	0.2540	11.90
irregular 10–40 μm MIP	287.6	0.3775	5.251
Silica (irregular 50–75 μm)	275.9	0.8362	12.12
irregular 50–75 μm NIP	76.70	0.2248	11.73
irregular 50–75 μm MIP	130.4	0.2903	8.904
Silica (irregular 75–150 μm)	252.2	0.3120	4.947
irregular 75–150 μm NIP	91.66	0.2176	9.495
irregular 75–150 μm MIP	108.5	0.2610	9.626

**Table 2:** Adsorption equilibrium time (t_e_ (min)), imprinting factor (IF) and equilibrium adsorption capacity (Q_e_ (mg·g^−1^)) of different MIPs on their corresponding supports.

Supports	t_e_ (min) *^a^*	IF *^a^*	Q_e_ (mg·g^−1^) *^b^*
Silica (spherical 0.5 μm)	40	3.28	65.5
Silica (spherical 5 μm)	40	2.65	51.4
Silica (spherical 60 μm)	10	2.28	92.9
Silica (irregular 10–40 μm)	30	1.64	84.5
Silica (irregular 50–75 μm)	40	1.41	68
Silica (irregular 75–150 μm)	50	1.50	62.2

***^a^*** t_e_ and IF of different MIPs were carried out in the condition: 10 mg of MIPs in 5 mL of 110 mg·L^−1^ gossypol solution. ***^b^*** Q_e_ was carried out in the condition as follows: 10 mg of MIPs in 5 mL of 200 mg·L^−1^ gossypol solution.

**Table 3:** Kinetic parameters for the adsorption of gossypol onto MIPs and NIPs prepared on 60 μm spherical silica.

		Pseudo-First-Order			Pseudo-Second-Order		
sample	Q_e,exp_ (mg·g^−1^)	Q_e,cal_ (mg·g^−1^)	k_1_ (min^−1^)	R^2^	Q_e_ (mg·g^−1^)	k_2_ (mg·g^−1^·min^−1^)	R^2^
MIP	54.3	53.67	0.6820	0.9994	54.30	0.07775	0.9999
NIP	23.8	22.36	0.2562	0.9669	24.27	0.01786	0.9917

**Table 4:** Langmuir and Freundlich isotherm model parameters for the MIPs and NIPs on 60 μm spherical silica.

		Langmuir			Freundlich		
sample	Q_e,exp_ (mg·g^−1^)	Q_m_ (mg·g^−1^)	K_L_ (L·mg^−1^)	R^2^	K_F_ (mg·g^−1^)	n	R^2^
MIP	204	185.9	0.03761	0.8416	50.81	4.979	0.9903
NIP	95	111.6	0.00484	0.9959	8.826	2.936	0.9542

**Table 5:** The adsorption capacity, imprinting factors (IF) and selectivity coefficients (α) of gossypol, ellagic acid and quercetin of MIP and NIP using spherical silica (60 μm) as the support.

Adsorbates	Q_MIP_ (mg·g^−1^)	Q_NIP_ (mg·g^−1^)	IF	α
Gossypol	92.9	42.3	2.20	-
Ellagic acid	48.7	43	1.13	1.94
Quercetin	16.8	14	1.20	1.83
